# The Seventeenth Annual Commencement of the Baltimore College of Dental Surgery

**Published:** 1857-04

**Authors:** 


*The Seventeenth Annual Commencement of the Baltimore College of Dental Surgery,* was held in the Saloon of the College Building, on the evening of the 27th of February. The valedictory of the occasion was delivered by Professor T. E. Bond. Of the class, which consisted of forty-five matriculants, twenty were admitted to the degree of Doctor Dental Surgery.

